# Idiopathic prepubertal unilateral gynecomastia: Case report and literature review

**DOI:** 10.1097/MD.0000000000017374

**Published:** 2019-10-04

**Authors:** Chenyu Wang, Nanze Yu, Lin Zhu, Ang Zeng

**Affiliations:** Division of Plastic and Aesthetic Surgery, Peking Union Medical College Hospital, Chinese Academy of Medical Sciences and Peking Union Medical College, Dongcheng Qu, Beijing Shi, China.

**Keywords:** prepuberty, therapy, unilateral gynecomastia

## Abstract

**Rationale:**

Prepubertal unilateral gynecomastia is extremely rare, whose etiology and management strategy are not familiar. We would like to present a case and a literature review of unilateral prepubertal gynecomastia.

**Patient concerns:**

A 11-year old male patient with complaints of unilateral enlargement of breast tissue presented in our clinic, whose physical examination, biochemical, hormonal and oncologic findings were normal.

**Diagnoses:**

This patient was diagnosed as idiopathic unilateral prepubertal gynecomastia (IUPG) and self-abasement, social isolation and sensitive of interpersonal relationship.

**Interventions:**

The patient received subcutaneous mastectomy. Histopathological examinations showed idiopathic gynecomastia of ductal epithelial hyperplasia and active interstitial fibrous hyperplasia, with no evidence of any pathological finding. Immunohistochemical examination showed estrogen receptor (ER)-α positive (70%), epidermal growth factor receptor (EGFR) positive, Her-2 positive (1+), Progesterone Receptor (PR) positive (80%).

**Outcomes:**

A remarkable improvement was observed both in the physical and mental conditions at the post-surgical 6-month follow-up visit, showing no evidence of recurrence.

**Lessons:**

Further investigation is needed to clarify the pathogenesis of IUPG. All patients with IUPG should have a full endocrine and oncologic evaluation, and surgical excision may be the individually designed for each patient with the help of MRI of breast.

## 1 Introduction

Gynecomastia is a disease with a character of a bilateral or unilateral enlargement of breast tissue in male subjects, which is mainly prevalent in the puberty and in males over age of 50 years.^[1–3]^ Gynecomastia in the puberty period is usually due to increased androgen levels and concurrent increase in conversion of androgens to estrogens,^[1,4,5]^ which is generally bilateral, physiological, and not necessary to receive surgery. However, prepubertal gynecomastia and especially idiopathic unilateral prepubertal gynecomastia (IUPG) is extremely rare and barely reported.^[1,6]^ Thus, we would like to present a case of IUPG with the treatment of subcutaneous mastectomy and a literature review of unilateral prepubertal gynecomastia. The patient and his parents have provided the informed consent for publication of the case.

## 2 Case report

**Figure 1:**
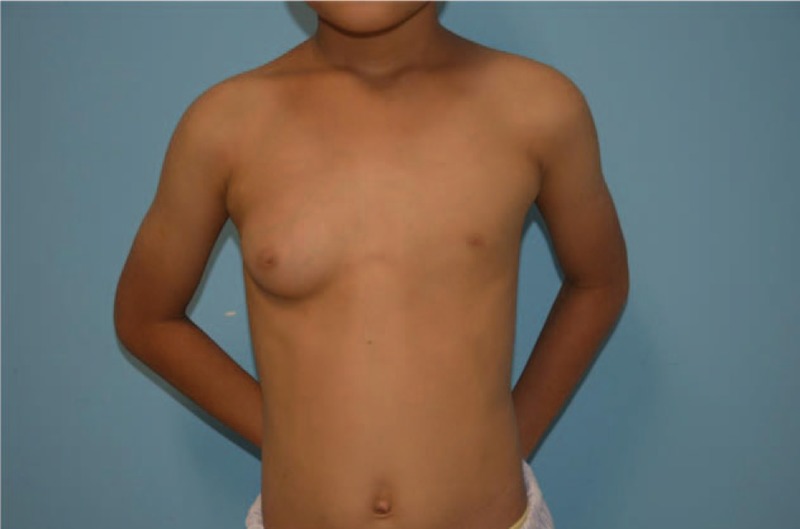
Unilateral gynecomastia before the surgery.

A 11-year-old boy presented to our clinic with enlargement of the right breast tissue of 4 years (Fig. 1). His medical and family history was unremarkable. At the time of admission, his height was 142 cm, his weight was 33 kg, and his BMI was 16.37 kg/m^2^. Physical examination showed he had breast development of 7cm × 7 cm on the right side and normal breast tissue on the left side. Both sides were without galactorrhea, nipple discharge, nipple retraction, lymphadenopathy, or skin changes, and no node was palpated. His testicles were 2cm × 1.5 cm bilaterally. Axillary and pubic hair stages were prepubertal.

**Figure 2:**
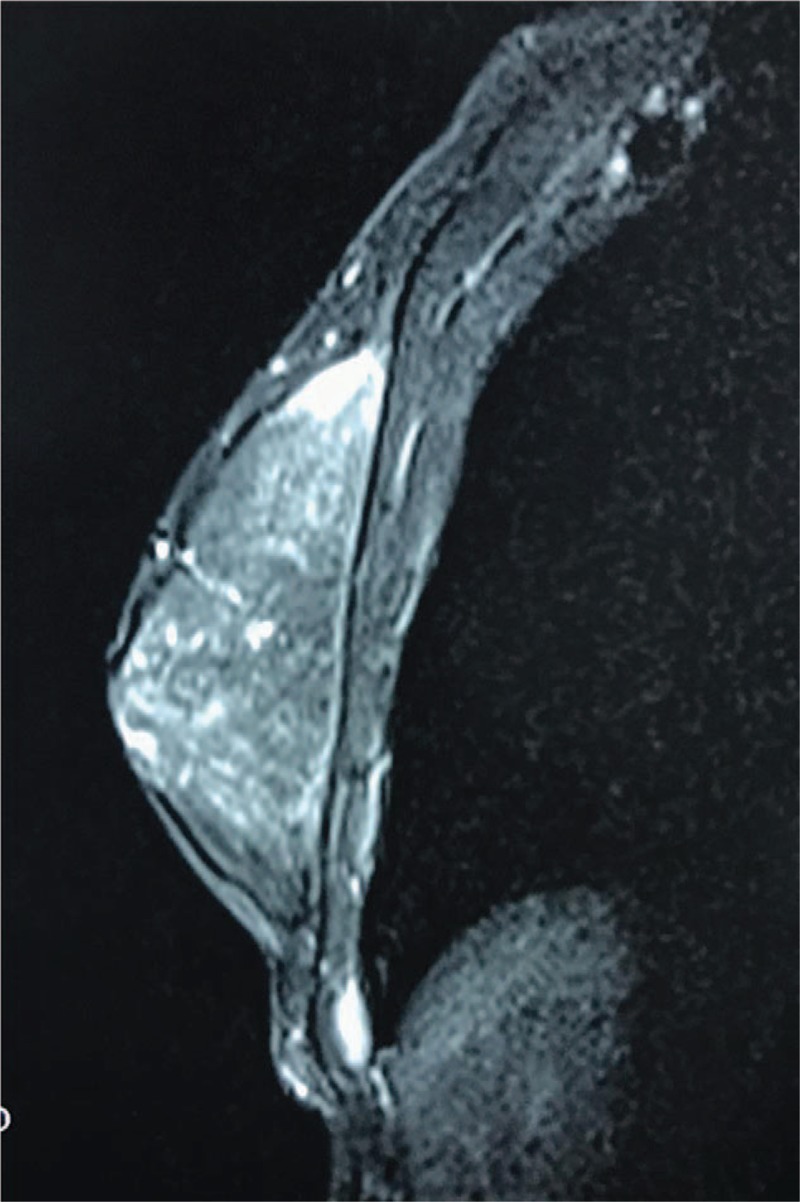
Magnetic resonance imaging (MRI) of unilateral gynecomastia (transversal plane, T1 weighted image).

**Figure 3:**
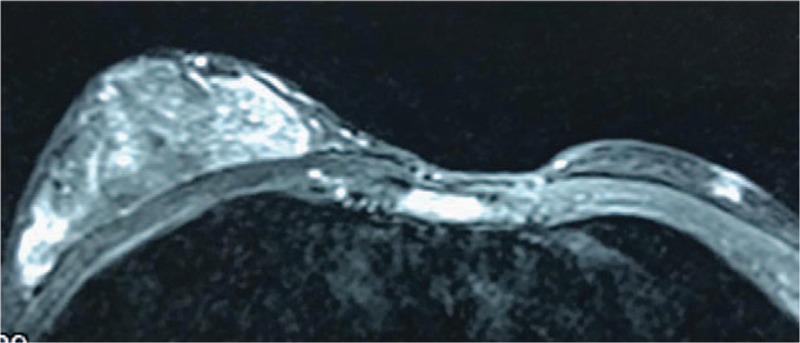
Magnetic resonance imaging (MRI) of unilateral gynecomastia (vertical plane).

Hormonal evaluation and oncological tests were normal (Table 1). Scrotal and abdominal ultrasound findings were normal. Breast ultrasound showed a fibroglandular tissue of 69 × 66 × 13.1 mm in the right side without any cystic or solid mass, and no findings in the left side. Magnetic resonance imaging (MRI) of breast showed hyperplasia of mammary glands on the right side (Figs. 2 and 3). Chromosome karyotype showed male karyotype of 46, XY. We also noticed that he had feelings of self-abasement, social isolation, and sensitive of interpersonal relationship due to concerns about his feminine breast appearance (12 scores, social anxiety scale for children; 6 scores, Achenbach child behavior checklist).

**Table 1:**
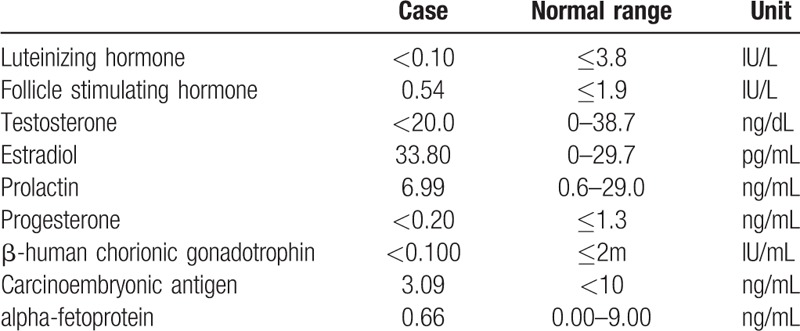
Hormonal evaluation and oncological tests of the patient.

### 2.1 Surgical method

**Figure 4:**
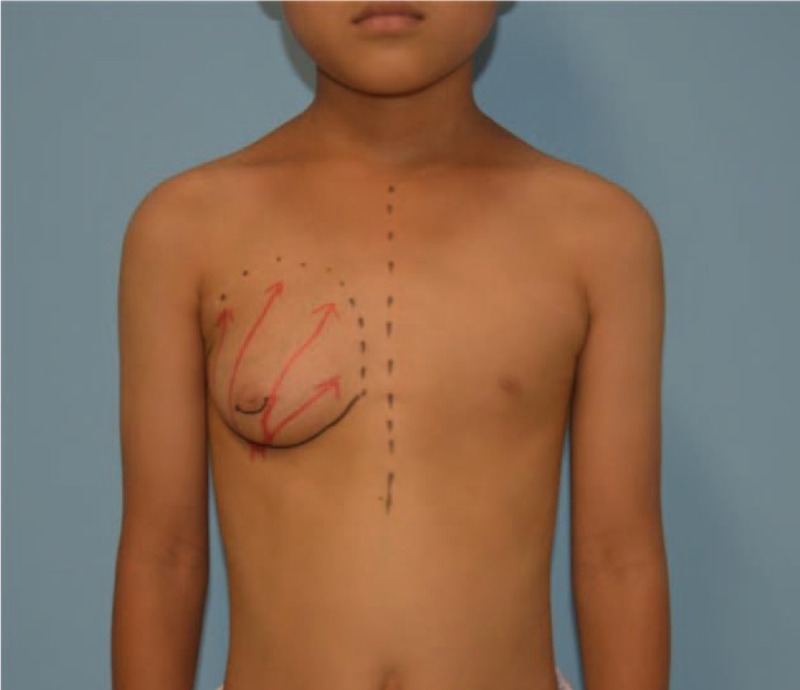
Unilateral gynecomastia before the surgery with mark.

**Figure 5:**
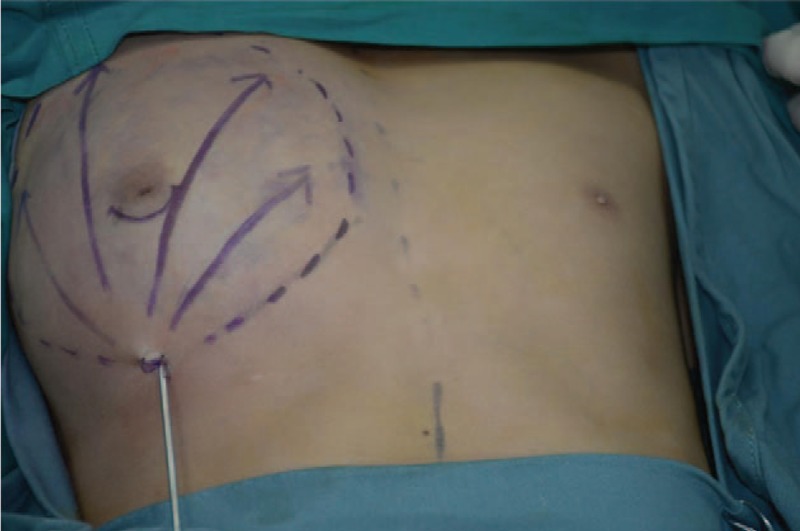
Unilateral gynecomastia during the surgery.

**Figure 6:**
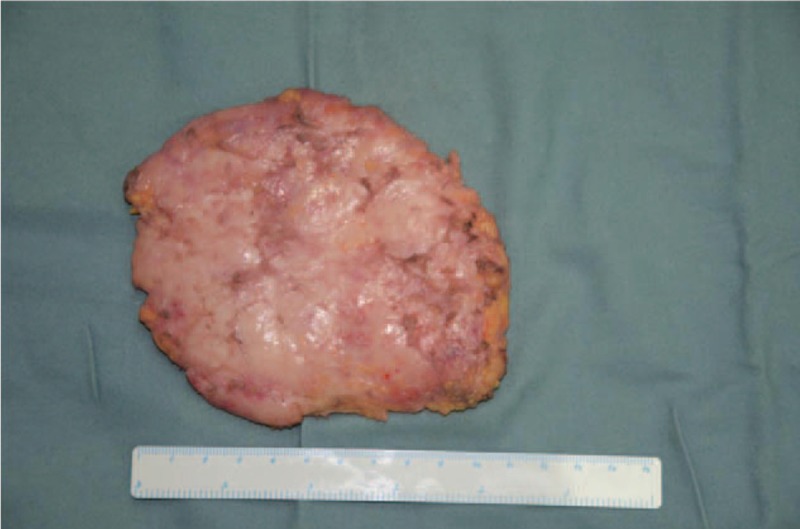
Excision tissue of the breast.

This patient was diagnosed as IUPG. Surgical resection was performed with peripheral liposuction at first (Figs. 4 and 5), finding that there was barely any fat to be sucked up. Then we performed the subcutaneous mastectomy through the incision along the inferior margin of the areolar, resecting the hypertrophic mammary tissues of 110 g (Fig. 6). The patient got discharged in 6 days after the surgery without perioperative complications.

**Figure 7:**
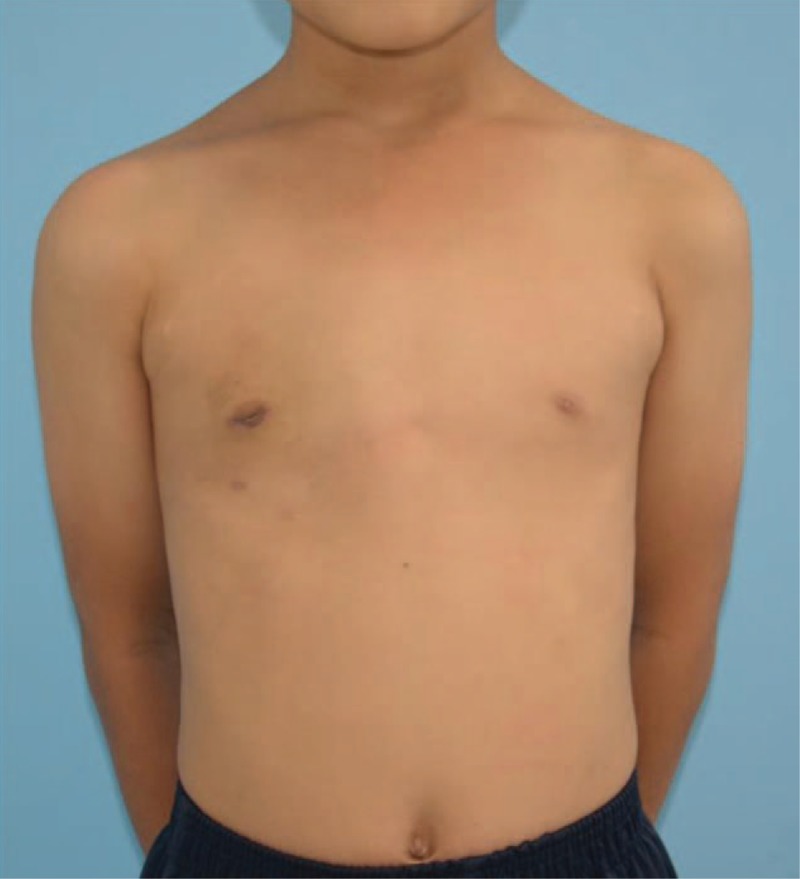
Six months after the surgery.

A remarkable improvement was observed both in the physical and mental conditions at the postsurgical 6-month follow-up visit, showing no evidence of recurrence (Fig. 7).

### 2.2 Pathological findings

**Figure 8:**
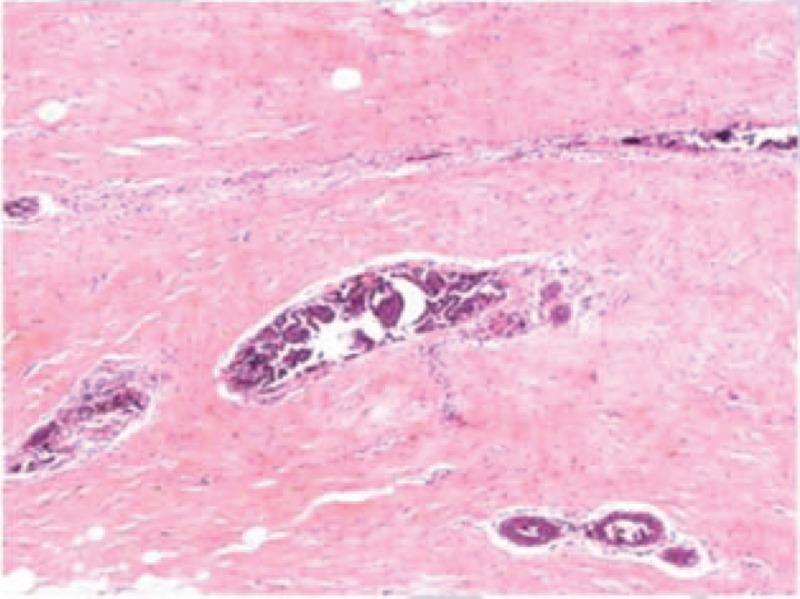
Histopathologic image of the excision tissue.

Histopathologic findings of the specimen showed idiopathic gynecomastia of ductal epithelial hyperplasia and active interstitial fibrous hyperplasia, with no evidence of any pathological finding (Fig. 8). Immunohistochemical findings showed CK14 (+), Syn (−), CK5/6 (+), estrogen receptor (ER)-α (strong positive,70%), epidermal growth factor receptor (+), Her-2 (1+), Ki-67 (index 5%), P53 (−), progesterone receptor (PR) (strong positive,80%), CgA(-)

## 3 Discussion

### 3.1 Literature review

To investigate more about Prepubertal unilateral gynecomastia, the PubMed, Embase, and Science Citation Index databases were systematically searched until October, 2018. The key words used were “Prepubertal unilateral gynecomastia”. Five articles with 5 case reported were enrolled (Table 2). Two cases were diagnosed as idiopathic unilateral prepubertal gynecomastia, 1 case was caused by pseudoangiomatous stromal hyperplasia, 1 case was due to the treatment of methylphenidate on attention deficit hyperactivity disorder, and 1 case was sex chromosome abnormality. Immunohistochemical results showed intensive ER on IUPG patients, while negative on other diseases.

**Table 2:**
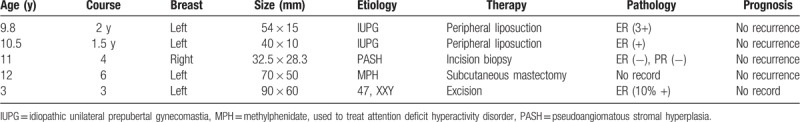
Literature review of prepubertal unilateral gynecomastia.

### 3.2 Etiology

The main mechanisms of gynecomastia, we believed, should be summarized as: increased estrogen levels due to endogenous excessive estrogen production or systemic exposure to exogenous estrogen; shift in the estrogen/androgen balance in favor of estrogen levels; increased estrogen sensitivity of the breast tissue; primary tumoral lesions or ono-mammary gland tissue hyperplasia diseases of breast tissue; medication side effect.^[1,3,4,7]^ Gynecomastia in the puberty period is usually due to increased androgen levels, or concurrent increase in conversion of androgens to estrogens, which is generally bilateral. While the mechanism of idiopathic unilateral prepubertal gynecomastia, as the evidence we had, is likely to be the overexpression of ERs on the unilateral breast tissue. The unilateral gynecomastia cases reported by Anderson^[3]^ and Demirbilek^[1]^ had presence of ER-positive results. In our case, serum estrogen and estrogen/testosterone levels were within the normal range, but the noteworthy presence of ERs (strong positive, 70%) and PR (strong positive, 80%) suggested increased local estrogen and progesterone sensitivity. The accurate reason for such phenomenon is still unknown and lack relevant report or research on it. From other reports, we noticed that the medication effect not only can appear on bilateral breast but also can present as UPG, which reminds us detailed history collection, including drug treatment history, is of great significance.^[8,9]^

### 3.3 Psychological influence

Compared with detailed research on psychological influence of pubertal gynecomastia patients, there is barely attention on the prepubertal. However, psychological influence of gynecomastia on children is far worse than we imagined. In our case, the young patient had already presented self-abasement, social isolation, and was sensitive of interpersonal relationship due to concerns about his feminine breast appearance. Esteem desire is the main pursuit of gynecomastia patients.

### 3.4 Diagnosis and therapy

Hormonal evaluation, liver and renal function tests, and oncological test should be carefully examined to evaluate the mechanisms of the gynecomastia before surgery.^[10,11]^ MRI of breast is highly recommended not just for diagnosis but also to distinguish the amount of the mammary gland, which is helpful for the choice of surgery procedure. Peripheral liposuction is usually taken to treat gynecomastia. However, patients with too many hypertrophic mammary tissues should be treated with subcutaneous mastectomy for better appearance recovery.

## 4 Conclusion

Further investigation is needed to clarify the pathogenesis of IUPG. All patients with IUPG should have a full endocrine and oncologic evaluation, and surgical excision may be the individually designed for each patient with the help of MRI of breast.

## Author contributions

**Conceptualization:** Chenyu Wang.

**Data curation:** Chenyu Wang.

**Formal analysis:** Chenyu Wang, Nanze Yu, Lin Zhu, Ang Zeng.

**Methodology:** Nanze Yu, Ang Zeng.

**Writing – original draft:** Chenyu Wang.

**Writing – review & editing:** Ang Zeng.
